# Patterns of Mammography Screening in Women with Breast Cancer in a Kansas Community

**Published:** 2023-02-12

**Authors:** S Lai, K Molnar, L Huang, S Garimella, M Enko, P Pordell, A White, V Senkomago

**Affiliations:** 1 Kansas Cancer Registry, Departments of Population Health, Orthopedic Surgery and Sports Medicine, University of Kansas Medical Center, Kansas City, Kansas, USA; 2 Kansas Cancer Registry, Department of Population Health, University of Kansas Medical Center, Kansas City, Kansas, USA; 3 Centers for Disease Control and Prevention, National Center for Chronic Disease Prevention and Health Promotion, Division of Cancer Prevention and Control, USA

**Keywords:** Breast Cancer Screening, Mammogram Screening Recommendations, Compliance

## Abstract

**Purpose:**

The objective of this study was to examine patterns of mammography screening prior to breast cancer diagnosis in all women with breast cancer in a Kansas community.

**Methods:**

The study population included 508 women in the Kansas Cancer Registry database diagnosed with breast cancer between 2013-2014 who were patients and residents of a defined area at the time of diagnosis. Screening history within 4 years of diagnosis was obtained. Poisson regression analysis was used to examine the relationship between sociodemographic factors and biennial screening.

**Results:**

About 41.5% of women received at least biennial screening, while 22.1% received less than biennial screening and 36.4% had no screening. About 40% of women aged 50-64, 50.4% aged 65-74, and 48.3% aged 75-84 received biennial screening (p=0.002). Women diagnosed with in-situ and localized breast cancers had significantly higher proportions of biennial screening (46.7% and 48.6%, respectively; p < 0.001). Average tumor size was 15.7, 17.4, and 24.4 mm, for women who received at least biennial, some, and no screening, respectively (p < 0.001). Results from Poisson regression analysis showed the adjusted relative risk associated with rural/mixed residence at diagnosis and Medicaid beneficiary was 0.45 and 0.40 (p=0.003 and p=0.032) respectively.

**Conclusions:**

Biennial mammography screening was associated with lower breast cancer stage and smaller tumor size, illustrating the importance of screening as early detection. Different outreach strategies may be necessary to reach women within varied age groups or geographical regions to help increase the number of women who remain up-to-date with mammography screening.

## Introduction

While researchers and public health partners recognize the observed improvement in early detection of breast cancer with use of mammography screening, the age-adjusted rates of breast cancers diagnosed at distant stage has not declined in the U.S [1-9]. According to United States Cancer Statistics (USCS), age-adjusted incidence rates for distant stage breast cancer among women were 6.4, 7.1, 7.5 and 7.1 per 100,000 in 2006, 2010, 2014, and 2018, respectively [10].

The published population-based data on mammography screening were mostly based on self-reported responses to national health surveys [11,12]. The 2018 Behavioral Risk Factor Surveillance System (BRFSS) report found that 66.6% of women surveyed previously had a mammogram, but 23.9% of those same women had not received a mammogram within the past 2 years. These surveys did not ascertain the history of breast cancer among women surveyed. Furthermore, there was no indication whether the mammogram was conducted for screening or diagnostic purposes. However, the 2018 National Health Interview Survey collected information to assess breast cancer screening per United States Preventive Services Taskforce (USPSTF) recommendations [13]. Among women aged 50-74 years, about 72% were up-to-date with mammography screening. However, respondents with a personal history of breast cancer were excluded from the analysis. There is a paucity of literature currently available that includes population-based data on mammogram screening history among women diagnosed with breast cancer.

Mammograms are generally acknowledged as an important tool for the early detection of breast cancer and there are differences among the major organizations on recommendations for screening frequency [14-17]. The American Cancer Society (ACS) recommends annual screening for women aged 45-54 years, transitioning to biennial screening beginning at age 55 and with screening to continue as long as a woman is in good health [14]. The National Comprehensive Cancer Network (NCCN) and The American College of Radiology (ACR) recommend annual screening mammograms beginning at age 40 [15,16]. The American College of Obstetricians and Gynecologists (ACOG) recommends women begin annual or biennial screening mammography at 40 years, with mammography initiation no later than 50 years, and continuing screening mammography until at least 75 years [17].

In 2009, the USPSTF recommended biennial screening mammography for 50 to74-year-old women. For women aged 40 to 49 years, the USPSTF stated “the decision to start regular biennial screening for women before the age of 50 years should be an individual one and take patient context into account, including the patient’s values regarding specifics benefits and harms” [18]. The USPSTF further concluded that the evidence at the time was insufficient to assess the balance of benefits and harm of screening mammography in women aged >75 years. Similar to the other organizations, USPSTF recommended at least biennial screening.

This study examined mammography screening in Kansas women who were diagnosed with breast cancer between 2013-2014. Given the variability of age and screening frequency in screening mammography recommendations, we chose to assess whether these women received mammography screening within four years of the primary breast cancer diagnosis and determined whether screening aligned with the 2009 recommendation from the U.S. Preventive Services Task Force (USPSTF) [18].

## Methods

### Study Cohort

The Kansas Breast Cancer Screening Study (KBCSS), a pilot project funded by the Centers for Disease Control and Prevention (CDC) National Program of Cancer Registries (NPCR) to assess the feasibility of breast cancer screening data collection and linkage by the state cancer registry, included 508 Kansas women who were diagnosed with breast cancer between 2013-2014 and were registered in the Kansas Statewide Cancer Registry (KCR) [19]. The 508 women were patients and residents of the KBCSS catchment area at the time of diagnosis. We selected this geographic region so that it would be similar to the population characteristics of Kansas and have the following attributes: 1) this catchment area has residents living in urban, rural, and mixed urban and rural settings; 2) the physical location is isolated enough to discourage residents from traveling a long distance for preventive care such as breast cancer screening; and 3) the region also has a complex medical care system including major medical centers, small sized hospitals, and clinics. This project was reviewed and approved by the University of Kansas Medical Center (KUMC) Institutional Review Board (IRB ID MOD00015076). Its conduct was in accordance with the Code of Federal Regulations (45 CFR 46) and State of Kansas Regulations and Administrations (K.S.A. 65-1, 168 – 174; K.A.R 28-70-1 to 28-70-4), which allowed KCR the authority to collect cancer screening data on study patients without informed consent [20,21].

This study focused on cases diagnosed in 2013 and 2014 for two reasons: 1) ascertainment of 2013 and 2014 diagnosed cases would be completed by 2018, the year the pilot project was funded; 2) collection of mammography screening within four years of breast cancer diagnosis allowed the KBCSS study to evaluate screening compliance according to the USPSTF 2009 recommendation; and 3) mammogram screening data, particularly data for women insured through Medicare, could be validated because claims data are available a few years after cancer diagnoses. Linkage with Medicare claims data allowed us to evaluate the extent to which women sought mammogram screening outside of their residence area. Additionally, our study validated breast cancer screening through a linkage between the KCR and Early Detection Work (EDW) program databases to ascertain breast cancer screening data for its participants. The EDW is part of CDC’s National Breast and Cervical Cancer Early Detection Program (NBCCEDP- https://www.cdc.gov/cancer/nbccedp/index.htm), which offers screening to women who have low incomes, are under- or uninsured, and have reduced access to breast and cervical cancer screening and diagnostic services [22].

### Identification of Mammography Screening

State and territorial central cancer registries are authorized to collect cancer-related information including data on cancer occurrence (including the type, extent, and location of the cancer), the type of initial treatment, and outcomes [23]. The previously stated existing Kansas legislation in cancer registration and the associated administrative regulations were revised to give KCR the authority to collect cancer screening data without informed consent [20,21]. Kansas enacted the updated legislation in December 2020.

To facilitate this project, the KBCSS study team developed a Microsoft Access database to collect mammogram related data items including date of mammogram screening and the mammogram results (left and/or right breast – normal or not normal; BI-RAD category, breast density, type of procedure – 2D, 3D, and billing codes if available in the report). We contacted all radiologists in the catchment area to assess their interest in participating in the KBCSS study. Radiologists confirmed that they submitted all mammogram reports to the catchment area hospitals. The mammogram reports pertaining to the study population were submitted to the KBCSS team by the hospitals for pertinent data abstraction. The KBCSS team also contacted hospitals outside the catchment area if there was information on women who had their mammograms at those hospitals.

We defined annual screening as women who received a screening mammogram in each of the four years prior to breast cancer diagnosis. The study team defined biennial screening as women who received a screening mammogram in alternate years or in three of the four years prior to diagnosis. We considered women who received only one mammography screening in the four years prior to diagnosis, or two screening mammograms in non-alternate years, as having less than biennial screening. Study investigators defined no screening as women who had not received a mammogram during the four years prior to diagnosis and women who only received one diagnostic mammogram prior to diagnosis.

The study team reviewed all mammogram reports and entered pertinent data into the project database. Because some diagnostic mammography screenings were considered as screening mammography due to reasons such as personal history/high risk, previous year’s screening results, or characteristics of breast tissue, manual reviews were conducted to reclassify diagnostic use of mammography to a screening status using all available mammography claims. For example, women who received screening mammography three years in a row and later received a diagnostic mammogram (or a series of diagnostic mammograms) around one year after the last screening mammography were reclassified as receiving an annual mammography screening instead of being defined as biennial. We also applied review and reclassification for cancer cases that were categorized as receiving biennial screening. Any classification discrepancies were resolved after study team discussion and consensus.

### Statistical Analyses

Patient demographics including age, race/ethnicity, insurance/payer information, stage at diagnosis, and rural/urban residence were obtained from the KCR database. Rural/urban residence was defined using Rural-Urban Commuting Area (RUCA) ZIP code approximation codes for all patients [24]. Insurance/payer was categorized as private (including Tricare/VA) with Tricare and VA being employment affiliated options; Medicaid; Medicare without supplements; Medicare with supplements; and no insurance/self-pay. We used U.S. Census data to acquire median household income for the catchment area [25].

Stage at diagnosis was classified by the SEER Summary Stage 2000 system as *in-situ, localized*, *regional*, *distant*, or *unknown* [26]. Tumor size was recorded in millimeters. Tumor size was reported as exact size or coded to 990 (< 1 mm), 995 (4 cm – 5 cm), 998 (diffuse), and 999 (unknown). Codes 990 and 995 were recoded as 0.5 mm (3 cases), and 45 mm (1 case) respectively. Associations between categorical variables and mammography screening status were examined using χ2 or the Fisher exact test when appropriate.

Differences in the tumor size between the three screening groups were analyzed using one-way ANOVA or the Wilcoxon rank test if normality was not held up. Poisson regression analysis was used to examine the relationship between the sociodemographic study variables and the level of mammography screening [27]. All significance tests were 2-sided at the .05 level. The Poisson assumption was confirmed. The study team used SAS version 9.4 software to conduct all analyses [28].

## Results

The catchment area had 508 primary female breast cancers that were diagnosed in 2013 and 2014. Ninety-three percent of those diagnosed with breast cancer were White and the remaining 7% were Black and Other races. Table 1 shows mammography screening by individual and community characteristics. A little over 40% of women aged 50-64 years, 50.4% of women aged 65-74, and 48.3% of women 75-84 years were compliant with the 2009 USPSTF mammography screening recommendation of at least biennial screening via mammogram (p=0.002, Table 1).

Forty-one of the 71 women younger than 50 years of age (57.8%) did not have any mammography screening in the four years prior to breast cancer diagnosis. Forty of the 71 women under 50 had a family history of breast cancer. About 44% of women living in urban areas received the 2009 USPSTF screening recommendation as opposed to 20% of women who resided in rural/mixed settings. A statistically significant difference was observed with those having Medicare insurance as payer at diagnosis. These women were more likely to be compliant (p=0.005). No statistically significant difference was noted in median household income.

Table 2 shows the association between mammography screening and tumor characteristics. Women diagnosed with in-situ and localized breast cancers had statistically significant higher proportions of receiving the 2009 recommended mammography screening (46.7% and 48.6%, respectively; p < 0.001). The average tumor size was 15.7 mm, 17.4 mm, and 24.4 mm, correspondingly for those who received recommended screening, some screening, and no screening (p < 0.001).

Results from the Poisson regression analysis of factors associated with compliance with the USPSTF recommendations are summarized in Table 3. The adjusted relative risk associated with the rural/mixed residence at diagnosis was 0.45 (95% CI: 0.26 – 0.76; p=0.003). The adjusted relative risk associated with Medicaid beneficiary was 0.40 (95% CI: 0.17 - 0.92; p=0.032).

## Discussion

To our knowledge, this study is the first U.S. population-based study assessing mammography screening history in all women diagnosed with breast cancer in a defined region of Kansas. Mammograms are generally acknowledged as an important tool for the early detection of breast cancer and there are differences among the major organizations on recommendations for screening frequency and the targeted age [14-17]. Our study examined whether women diagnosed with breast cancer received mammography screening in accordance with the USPSTF 2009 recommendation which did not include women aged < 50 and > 75 years with average risk of breast cancer [18]. About 45% (143/321) of women with breast cancer in the USPSTF recommended age group received at least biennial screening.

Surprisingly, 48.3% of women with breast cancer aged 75-84 years received at least biannual mammogram screening prior to their diagnosis. Our study explored whether a family history of breast cancer might explain why women younger than 50 years chose to receive mammogram screening or not. We found that 40 of the 71 women in this age group had a positive family history of breast cancer. Other major organizations such as ACS, NCCN, and ACR have recommendations for younger aged women, with ACS and ACR providing additional recommendations for high risk women [15-17]. A statement similar to the ACR recommendation encouraging women in this age group to receive screening if they have a calculated lifetime breast cancer risk of 20% or greater based on their family history may be warranted [29].

Our study also showed women with breast cancer residing in rural/ mixed settings had a 55% less chance of receiving biennial screening in the four years preceding their breast cancer diagnosis. The proportions of women receiving the recommended mammogram screening were similar between non-White and White Kansas women with breast cancer. Despite a similarity in the racial distribution between the study area and Kansas, the number of non-White female breast cancer cases in the study area is too small for a meaningful interpretation of the data.

As noted earlier, tailored strategies may be warranted based on geographic residence as a way to improve the current rate of mammogram screening among those in the age groups recommended by USPSTF. Two published studies examined screening mammography based on female breast cancers diagnosed at a single hospital [30,31]. Moorman et al (2021) identified 490 patients aged 40-84 with breast cancer during 2016-2017 from a single hospital. Fifty percent of the patients (245/490) had undergone annual, biennial, or triennial screening [30].

About 41% (200/490) had annual screening, followed by 6.5% and 2.7% biennial and triennial screening, respectively. Another single institution study by Ahn et al (2018) included 1,125 patients aged > 40 years with breast cancer diagnosed from September 2008 to May 2016 [31]. Seventy-three percent had screening between 1–24 months before diagnosis, 21% had screening at 25+ months, and 6% never had mammography. The definition of screening mammograms was not described in either study.

Interestingly, our population-based study found that 42% of women followed the 2009 USPSTF recommended timeline of mammogram screening as opposed to 50% in a pool of hospital-based women with breast cancer [30]. Our study included 2013 to 2014 diagnosed breast cancer cases while Moorman’s study had cases diagnosed during 2016-2017. The rate of mammogram screening compliance in our study is similar to that of the study by Moorman et al.

Our study analyzed all breast cancer cases in a defined geography, instead of focusing solely on hospital-based cases. Additionally, the distribution of stage at diagnosis in our study region is similar to that of Kansas and the U.S. for the 2013 and 2014 diagnosis years.

Our study observed several challenges in assessing level of compliance to the recommended mammogram screening recommendation. A minimum of three consecutive years of screening allowed us to have a clearer picture of a woman’s screening history and compliance to screening recommendations before diagnosis of cancer. Our unique findings in screening compliance may be useful to breast cancer screening advocates and/or educators at community and state levels.

Another challenge may be related to an individual’s health care seeking behavior and the complexity of our health care delivery systems. Many patients visited health professionals with different specialties that were not within a health system. As such, medical records were maintained by different providers, making access to complete medical records and consolidation of screenings and treatments difficult. Linkage with Medicare claims data allowed us to evaluate the extent to which women sought mammogram screening outside the study area.

Claims from four women in the study would have changed their screening status from no screening to two with at least biennial screening and two with less than biennial screening. However, Medicare only provides the mammogram procedural codes and not the reports themselves. Without access to the reports to verify the codes, we could not update the mammography screening for these women. Another challenge is related to outpatient providers’ unwillingness to disclose negative mammogram reports to a cancer registry due to patient’s confidentiality, though this is critical to the evaluation of screening compliance.

Data linkages with hospitals using procedural codes would have reduced a substantial amount of personnel time in reporting facilities and central cancer registries. With information on the mammogram reports currently not being extractable, it is extremely challenging for state cancer registries to take on collecting and reporting screening-related data in addition to cancer incidence registration and consolidation responsibilities.

Lastly, personnel assigned to collect screening data may be a concern for hospitals with competing priorities to maintain operations. This additional data collection may add to a facilities’ financial burden to recruit additional abstractors. Moreover, facility reporters/certified tumor registrars must be trained in reviewing mammogram reports with data items such as differences in screening versus diagnostic mammogram procedural codes, BI-RAD category, etc. The workflow in collection of screening data may conflict with the workflow related to abstraction of cancer incidence data.

## Conclusion

Routine mammography screening has been recommended for improving early detection of breast cancer in women. However, some populations may still be disproportionately affected. These findings differ greatly by age group, with women aged 65-74 and 75-84 years having the highest level of compliance, despite the latter age group not being subject to the recommendations. The adoption of recommendations may not be implemented as intended. Non-urban (rural/mixed) residency and Medicaid beneficiaries had statistically significant lower screening compliance.

These findings underscore the need to continue promoting breast cancer screening. This includes implementing different strategies to reach women of different backgrounds. Collection of cancer screening information by state central cancer registries is vital, but requires additional funding, as cancer registries may not have the staffing, time, or infrastructure required to implement processes and revise surveillance systems to collect cancer screening data.

## Figures and Tables

**Table 1:** Mammography screening by demographics and socio-economic status: Kansas Breast Cancer Screening Study (N=508).

Characteristics	Total	2009 USPSTF screening not compliant		2009 USPSTF screening compliant^2^ minimal biennial n=211	
		No screening (n=185)	Some screening <biennial^1^ n=112		
	N	Count (Row %)	Count (Row %)	Count (Row %)	p-value
Age at diagnosis (yrs)					0.002
<50	71	41 (57.8)	14 (19.7)	16 (22.5)	
50-64	182	62 (34.1)	47 (25.8)	73 (40.1)	
65-74	139	40 (28.9)	29 (20.9)	70 (50.4)	
75-84	89	31 (34.8)	15 (16.9)	43 (48.3)	
> 85	27	11 (40.7)	7 (25.9)	9 (33.3)	
Race/Ethnicity					0.424
White (Non-Hispanic)	462	165 (35.7)	99 (21.4)	198(42.9)	
White (Hispanic)	11	6 (54.5)	2(18.2)	3(27.3)	
Black	25	10 (40.0)	7 (28.0)	8 (32.0)	
Other	10	4 (40.0)	4 (40.0)	2 (20.0)	
Residence^3^					<0.001
Urban	459	152 (33.1)	106 (23.1)	201 (43.8)	
Rural/Mixed	49	33 (67.4)	6 (12.2)	10 (20.4)	
Median household income^4^					0.155
< $52,900	46	20 (43.4)	13 (28.3)	13 (28.3)	
>= $52,900	462	165 (35.7)	99 (21.4)	198 (42.9)	
Insurance payer at diagnosis					0.005
Medicaid	27	19 (70.4)	4 (14.8)	4 (14.8)	
Medicare without supplements	167	54 (32.3)	35 (21.0)	78 (46.7)	
Medicare with supplements	77	23 (29.9)	14 (18.2)	40 (52.0)	
No insurance/self-pay	34	15 (44.1)	7 (20.6)	12 (35.3)	
Private/Tri-care/VA	203	74 (36.5)	52 (25.6)	77 (37.9)	

1 Received one or two mammograms not according to the recommended timeline

2 Met annual or biennial mammogram screening recommendation

3 Rural-urban commuting area (RUCA) based on U.S. census tracts using measures of population density, urbanization, and daily commuting

4 KBCSS study region specific data from the Demographic Statistical Atlas of the United States - Statistical Atlas

**Table 2:** Mammography screening by tumor characteristics: Kansas Breast Cancer Screening Study (N=508).

Characteristics	Total	2009 USPSTF screening not compliant		2009 USPSTF screening compliant^2^	
		No screening	Screening frequency not-compliant^1^		
	n	Count (Row %)	Count (Row %)	Count (Row %)	p-value^3^
	508	175 (34.4)	112 (22.1)	211 (41.5)	
Stage at diagnosis					<0.001
In-Situ	75	21 (28.0)	19 (25.3)	35 (46.7)	
Local	290	86 (29.7)	63 (21.7)	141 (48.6)	
Regional	119	65 (54.6)	25 (21.0)	29 (24.4)	
Distant	21	11 (52.4)	4 (19.0)	6 (28.6)	
Unknown	3	2 (66.6)	1 (33.4)	0 (0.0)	
Tumor size (mm)^4^					
Mean +/− SD	14.6	24.4 +/− 22.69	17.4 +/− 14.01	15.7 +/− 13.79	<0.001^4^
Median	15	20	14	12	<0.001

1 Received one or two mammograms not according to the recommended timeline

2 Met annual or biennial mammogram screening recommendation

3 p-value was calculated using Chi-square test except tumor size. Unknown was included in the test.

4 A mid-value imputed for those tumor size code 900 (less than 1mm) and 995 (between 4 cm and 5 cm). Unknown tumor size (999) and diffuse (998) were not included in calculation of level of statistical significance (n=31).

**Table 3:** Factors associated with compliance with the U.S. Preventive Services Task Force recommended annual/biennial mammography screening: Results from the Poisson regression analysis (N=508).

Factors	Adjusted Relative Risk*	95% CI	P value
Age			
<50	0.86	0.40 – 1.88	0.7093
50-64	1.45	0.74 – 2.86	0.2827
65-74	1.55	0.86 – 2.77	0.1421
75-84	1.52	0.83 – 2.78	0.1739
>85	Reference	-	-
Non-white	1.06	0.36 – 3.12	0.9220
Rural/mixed residence at diagnosis	0.45	0.26 – 0.76	0.0032
Median household income	1.34	0.51 – 3.52	0.5518
Payer/insurance			
Medicaid	0.40	0.17 – 0.92	0.0324
Medicare without supplements	1.08	0.69 – 1.69	0.7266
Medicare with supplements	1.20	0.56 – 1.59	0.4558
No insurance or self-pay	0.94	0.56 – 1.58	0.8300
Private/Tri-care/VA	Reference	-	-
